# Supplementary material for: The causal relationship between the human gut microbiota and pyogenic arthritis: a Mendelian randomization study
Source: Front Cell Infect Microbiol. 2024 Nov 26;14:1452480. doi: 10.3389/fcimb.2024.1452480 (PMC11629706; doi:10.3389/fcimb.2024.1452480)
Supplement: Supplementary file 1 [file DataSheet1.zip › mendelian test/ebi-a-GCST90027795.csv_forest.pdf]

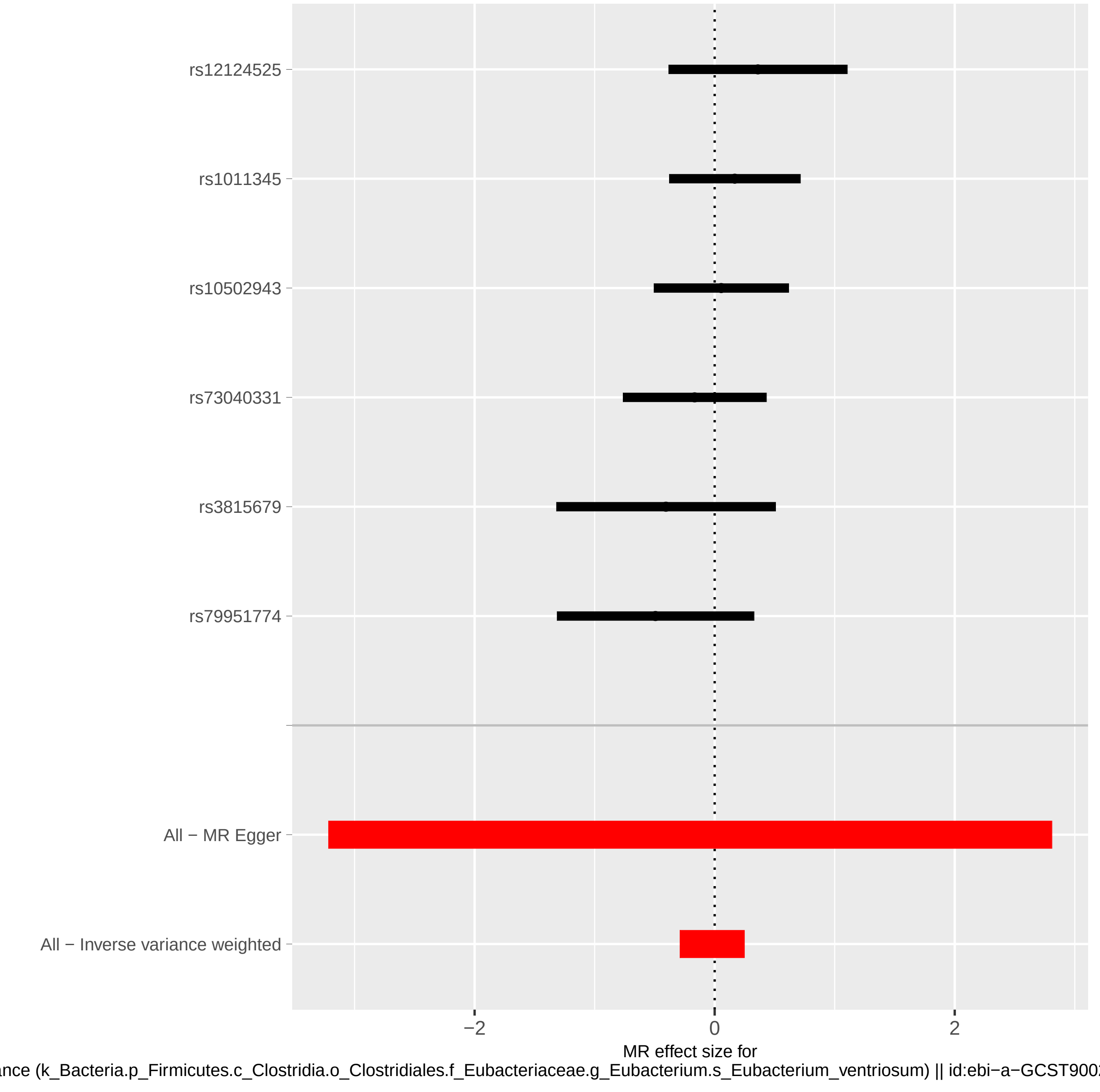

rs12124525
rs1011345
rs10502943
rs73040331
rs3815679
rs79951774
All − MR Egger
All − Inverse variance weighted
−2
0
2
MR effect size for
ance (k_Bacteria.p_Firmicutes.c_Clostridia.o_Clostridiales.f_Eubacteriaceae.g_Eubacterium.s_Eubacterium_ventriosum) || id:ebi−a−GCST900
